# Disentangling the roles of cholesterol and CD59 in intermedilysin pore formation

**DOI:** 10.1038/srep38446

**Published:** 2016-12-02

**Authors:** Courtney M. Boyd, Edward S. Parsons, Richard A. G. Smith, John M. Seddon, Oscar Ces, Doryen Bubeck

**Affiliations:** 1 Department of Life Sciences, Sir Ernst Chain Building, Imperial College London, London SW7 2AZ, UK; 2 Department of Chemistry and Institute of Chemical Biology, Imperial College London, London SW7 2AZ, UK; 3 London Centre for Nanotechnology, University College London, London WC1H 0AH, UK; 4 MRC Centre for Transplantation, King’s College London, 5th Floor Tower Wing, Guys’ Hospital, London SE1 9RT, UK

## Abstract

The plasma membrane provides an essential barrier, shielding a cell from the pressures of its external environment. Pore-forming proteins, deployed by both hosts and pathogens alike, breach this barrier to lyse target cells. Intermedilysin is a cholesterol-dependent cytolysin that requires the human immune receptor CD59, in addition to cholesterol, to form giant β-barrel pores in host membranes. Here we integrate biochemical assays with electron microscopy and atomic force microscopy to distinguish the roles of these two receptors in mediating structural transitions of pore formation. CD59 is required for the specific coordination of intermedilysin (ILY) monomers and for triggering collapse of an oligomeric prepore. Movement of Domain 2 with respect to Domain 3 of ILY is essential for forming a late prepore intermediate that releases CD59, while the role of cholesterol may be limited to insertion of the transmembrane segments. Together these data define a structural timeline for ILY pore formation and suggest a mechanism that is relevant to understanding other pore-forming toxins that also require CD59.

Pore-forming proteins oligomerize on target cell membranes to punch holes in lipid bilayers. Cytotoxic pores can be used for either attack or defense and are prolific throughout all kingdoms of life^1^. Pore-forming toxins represent the largest group of virulence factors secreted by pathogenic bacteria^2^. Produced by both Gram-positive and Gram-negative bacteria^3^, cholesterol-dependent cytolysins (CDCs) comprise a subset of toxins that require cholesterol to form giant β-barrel pores in lipid bilayers^4^. Despite variations in size and stoichiometry that make up mature pore complexes, the general mechanism of pore formation is highly conserved. The process is initiated when soluble toxin monomers bind to their target membrane^5^. Membrane-binding allosterically activates the monomer and promotes oligomerization^6^. Finally, the complex undergoes dramatic structural rearrangements to form the transmembrane pore^7^.

Structural analyses of soluble CDC monomers have defined a highly conserved modular arrangement of four domains^8^,^9^,^10^. Domains 1 and 3 (D1 and D3) make up the Membrane Attack Complex/Perforin-like Fold (MACPF), a kinked ‘L’-shaped motif formed by a central β-sheet. Upon pore formation, α-helical bundles within D3 unfurl and contribute two β-hairpins to the transmembrane pore^11^,^12^. In contrast, Domain 4 (D4) governs membrane-binding and cholesterol recognition^13^. In the soluble toxin, transmembrane segments in D3 are located far above D4 membrane-interacting residues (~30–40 Å). Therefore, to traverse the bilayer CDCs must undergo a vertical collapse and structural rearrangement involving Domain 2 (D2), an elongated and twisted β-sheet^14^.

For many CDCs, cholesterol-binding is sufficient to trigger conformational changes in the toxin during pore formation; however a sub-class, for which intermedilysin (ILY) is an archetypal member, also require the immune receptor CD59^15^. ILY is secreted by *Streptococcus intermedius* and is the major virulence factor for the bacterium associated with the formation of brain and liver abscesses in human hosts^16^. ILY’s specificity for human cells is conferred through its interaction with CD59, a small glycophosphatidyl-inositol (GPI)-anchored protein that inhibits pore formation of the complement membrane attack complex^17^. Mutational analyses and structural studies of the ILY-CD59 complex have demonstrated that although the CD59-binding site is located in D4, residues that make up the interface are different from those that interact with cholesterol in the lipid bilayer^18^,^19^. While it is known that both cholesterol and CD59 must be present for ILY to permeate the target cell, the precise role of each receptor remains unclear.

Here we use model membrane systems decorated with CD59 and conformationally-locked ILY variants to disentangle structural transitions triggered by cholesterol and human CD59. Specifically, we investigate the roles of each receptor in ILY oligomerization, vertical collapse of the prepore complex, and membrane lysis. Adopting a dual biochemical and biophysical approach, we find that CD59 is required for coordinating ILY monomers into an oligomeric prepore that can collapse towards the membrane. Formation of an SDS-resistant late prepore depends on structural transitions enabled by a movement between D2 and D3. Our data suggest that CD59 is released from the late-prepore, and that cholesterol triggers the final stages of membrane insertion.

## Results

ILY requires both cholesterol and CD59 to form pores in lipid bilayers. To distinguish the roles of these two receptors in structural transitions of pore formation, we developed a versatile model membrane system whose lipid composition could be altered and that incorporated soluble CD59 modified to contain a myristolated lysine-rich “cytotopic” peptide (^cyto^CD59)^20^. This system was adapted to a variety of model membranes including liposomes, monolayers and supported lipid bilayers for use in biochemical assays and imaging by electron microscopy (EM) as well as atomic force microscopy (AFM) techniques.

### ILY oligomerization

**Figure 1:**
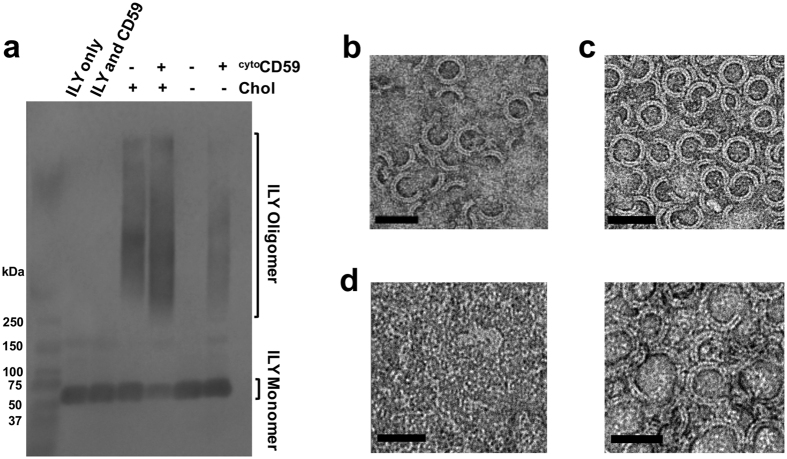
CD59 coordinates a specific geometry for ILY oligomeric prepores. (**a**) Wildtype ILY was incubated with CD59-decorated liposomes; monomers and oligomers were separated by SDS-AGE and transferred to nitrocellulose. His-tagged toxin was detected using western blot analysis. The + or − denoted at the top of the panel indicates whether the toxin was incubated with or without cholesterol-containing liposomes (*Chol*) or ^cyto^CD59. *ILY only* contains neither liposomes nor CD59. *ILY and CD59* refer to the incubation of soluble proteins in the absence of membranes. Molecular weight marker is in the far left lane. (**b**–**d**) Electron micrographs of negatively stained ILY oligomers formed on lipid monolayers. (**b**) ILY-^cyto^CD59 complexes on cholesterol containing monolayers. (**c**) ILY-^cyto^CD59 complexes formed on cholesterol-lacking monolayers. (**d**) ILY oligomers on cholesterol-rich monolayers formed in the absence of CD59. Two types of oligomers are shown in left and right panels. Scale bar, 50 nm.

CD59 is known to induce the formation of sodium dodecyl sulphate (SDS)-resistant ILY oligomeric pores on the surface of human cells^21^. To verify that our model membrane system could also support the formation of SDS-resistant oligomers, consistent with late-prepore and pore states of ILY, we used Agarose Gel Electrophoresis (AGE) followed by western blot analysis (Fig. 1a). In the absence of membranes, neither ILY alone nor ILY with soluble CD59 could form oligomers. In addition, ILY remained monomeric when incubated with liposomes lacking both cholesterol and ^cyto^CD59. Furthermore, SDS-resistant oligomers formed on membranes containing both cholesterol and ^cyto^CD59. However, unexpectedly oligomers were also observed on liposomes when either of these two components was lacking. Electron microscopy of negatively stained monolayers was used to visualize ILY oligomers (Fig. 1b–d). Independent of cholesterol in the lipid composition, all monolayers containing ^cyto^CD59 possessed regular ring and arc-like geometries (Fig. 1b–c and Supplementary Table S1), consistent with previously reported CDC pores^7^. On cholesterol-containing monolayers lacking ^cyto^CD59, ILY was mostly found to form nonspecific aggregates. However, densely packed irregular rings were also seen in some areas of the monolayers where local concentrations of cholesterol may have been higher (Fig. 1d and Supplementary Table S1). Therefore, our biochemical and electron microscopy data together suggest that although ILY can form oligomers on cholesterol containing membranes, CD59 is involved in setting the specific template geometry that governs the diameter of oligomeric ILY rings.

### CD59 is responsible for collapse of the ILY prepore

**Figure 2:**
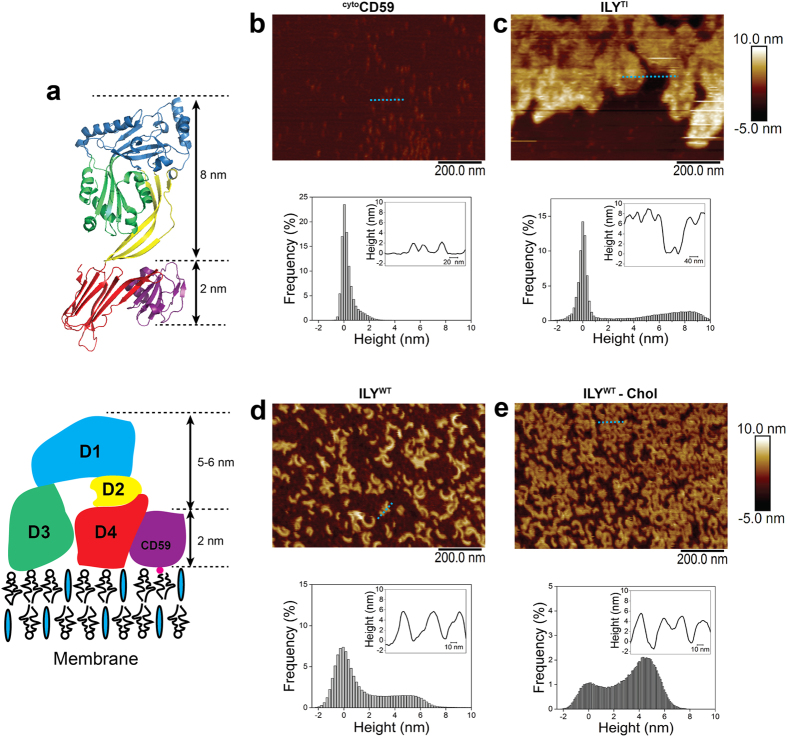
CD59 triggers a collapse of the ILY prepore. (**a**) Schematic of ILY structural transitions. ILY colored by domains (D1, blue; D2, yellow; D3, green; D4, red) and CD59 in purple. Top panel denotes the ILY^TI^-CD59 crystal structure (PDB ID: 4BIK)^19^. Bottom panel illustrates a model for the complex after collapse. (**b**–**e**) AFM images (top panel) and corresponding height data (bottom panel) for ILY-CD59 complexes on supported lipid bilayers. Height data is plotted as a histogram of the z measurements for each pixel in the image, with the inset showing a cross-section of the area highlighted by the blue dotted line. All bilayers contain cholesterol unless otherwise indicated by *–Chol*. Color scale, 15 nm. (**b**) ^cyto^CD59 on a supported lipid bilayer. (**c**) ILY^TI^ bound to a ^cyto^CD59-decorated bilayer. (**d**) wildtype ILY bound a to ^cyto^CD59-decorated bilayer. (**e**) Wildtype ILY bound to a ^cyto^CD59-decorated bilayer lacking cholesterol.

Upon pore formation, CDC oligomers undergo a dramatic vertical collapse bringing residues in D3 that penetrate the lipid bilayer close to their target membrane^14^. To test if ILY oligomers formed in the absence of cholesterol undergo a vertical collapse, we imaged complexes using AFM on supported lipid bilayers (Fig. 2). Previous studies have shown that forming large plaques of mobile prepores slow their diffusion and enable AFM imaging^7^. As such, ^cyto^CD59 was used to densely coat the membrane and restrict movement of ILY prepores. Moreover, baseline measurements read 2 nm above the membrane surface, consistent with the dimensions of CD59 (Fig. 2a,b). An early prepore intermediate of ILY captured with a disulfide-locked mutant (ILY^TI^) was used to assess the initial height of the complex. This mutant is unable to form SDS resistant oligomers or lyse cells; however, activity is restored under reducing conditions^21^. The ^cyto^CD59-bound ILY^TI^ prepore forms large, non-specific aggregates on monolayers (Supplementary Fig. S1) and supported lipid bilayers (Fig. 2c). The complex measures approximately 8 nm above the baseline, (10 nm above the membrane surface) (Fig. 2a and c), consistent with the ILY^TI^-CD59 crystal structure^19^ and in agreement with other CDC prepore structures^7^,^14^. In contrast, wild-type ILY oligomers that bound ^cyto^CD59-containing bilayers were between 5 and 6 nm above the baseline (7–8 nm above the membrane), independent of cholesterol in the membrane composition (Fig. 2d,e and Supplementary Fig. S2). To test if cholesterol alone could trigger the vertical collapse of oligomers, non-specific aggregates and clustered, irregular rings formed in the absence of ^cyto^CD59, similar to those observed on negatively stained monolayers, (Fig. 1d) were measured by AFM (Supplementary Fig. S3). These irregular oligomers measured between 10 and 15 nm above the membrane surface. Together, these data suggest the structural transitions that result in a vertical collapse of ILY are mediated by CD59 and not cholesterol.

### CD59 release from ILY prepore states

**Figure 3:**
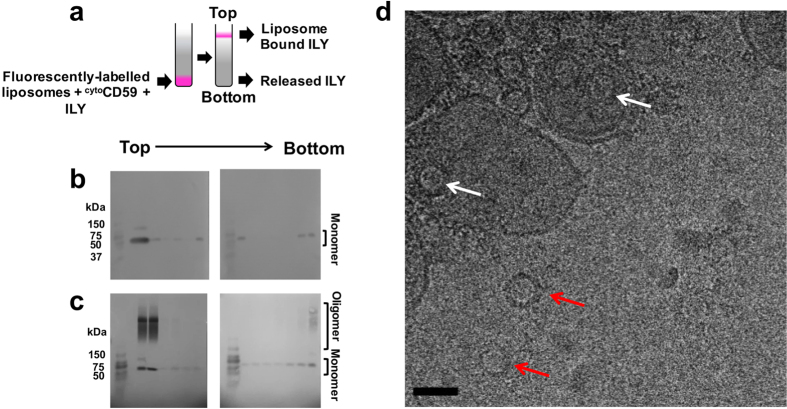
CD59 releases an ILY late-prepore independent of cholesterol. (**a**) Schematic illustrating the liposome flotation assay. Liposomes decorated with ^cyto^CD59 were incubated with ILY and subjected to density centrifugation. Monomeric and oligomeric forms of the toxin in each fraction were separated by SDS-AGE and detected by western blot analysis. Samples containing cholesterol-rich liposomes and the ILY^TI^ variant are shown in (**b**), while those in (**c**) contain wildtype ILY prepores formed on liposomes lacking cholesterol. Fractions from a single gradient span two gels (left and right). Directionality of fractionation is indicated by *Top* and *Bottom* above gels. The first lane in each gel contains molecular weight markers. (**d**) Cryo-electron micrograph of the sample analyzed in panel *c* showing ILY oligomeric prepores on liposomes (white arrows) and those released from the membrane (red arrows). Scale bar, 50 nm.

ILY binds CD59 during the early stages of pore formation; however, the receptor is released from the final transmembrane complex^21^. To define the temporal relationship between oligomerization, vertical collapse, and CD59-release, we used a flotation assay to assess ILY co-localization with CD59-decorated liposomes (Fig. 3a). Rhodamine-labeled phosphatidylethanolamine, included in the lipid composition, was used to track membrane-containing fractions across a ficoll density gradient. Nearly all ^cyto^CD59 present in the reaction associated with liposomes (Supplementary Fig. S4); any remaining soluble CD59 is unable to cause oligomerization (Fig. 1a). ILY^TI^, a variant unable to undergo a vertical collapse (Fig. 2c), was incubated with ^cyto^CD59-decorated liposomes and subjected to density centrifugation (Fig. 3b). Western blot analysis of AGE gel fractions showed that ILY^TI^ prepore complexes were SDS-sensitive, in agreement with previous studies^21^, and that the majority of ILY^TI^ colocalized with lipid-containing fractions. Some ILY^TI^ remained at the bottom of the gradient, consistent with a dynamic binding interaction of transient early prepore complexes^22^. To test for the release of late prepores, ^cyto^CD59 decorated liposomes that lacked cholesterol were incubated with wild type ILY (Fig. 3c). Top and bottom fractions of the gradient contained SDS-resistant oligomers, indicating that CD59 can release a late-prepore conformation. As soluble proteins do not spontaneously oligomerize even at high concentrations (Fig. 1a), non-membrane associated oligomers must be formed on the bilayer prior to release. The presence of oligomeric ring structures on and off liposomes was confirmed by 2D electron cryo-microscopy (cryo-EM) (Fig. 3d). While oligomers released from the membrane clearly dissociate from CD59, those that remain could either be still bound to CD59 or interact with membranes in a CD59-independent manner. It is possible that trans-membrane segment (TMS) regions, unrestricted in wildtype ILY, could unfurl and interact with the surface of the bilayer in a transient manner whereby the affinity of the interaction is insufficient to maintain membrane contact. Monomeric ILY was also observed in both regions of the gradient. These data suggest that oligomer formation maybe less efficient in the absence of cholesterol. As pores generate large holes in liposomes, resulting in vesicles filled with ficoll, wildtype pore conditions were unable to be characterized by flotation. However, previous cryo-EM studies of wildtype pores demonstrate that oligomers remain membrane bound^19^.

**Figure 4:**
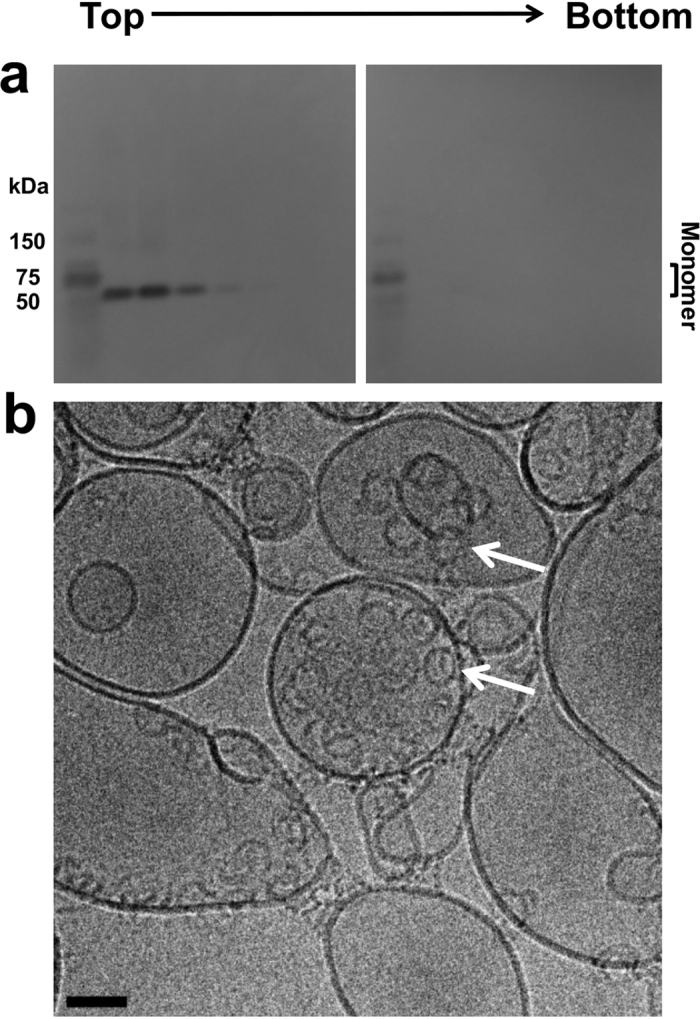
ILY^IG^ forms an SDS-sensitive early prepore that remains membrane-bound. (**a**) ILY^IG^ was incubated with ^cyto^CD59-decorated, cholesterol-containing liposomes and subjected to flotation through ficoll. Toxin within gradient fractions was analyzed by SDS-AGE and western blot. Fractions span two gels with direction of fractionation indicated by *Top* and *Bottom*. Molecular weight markers are in the first lane of each gel. (**b**) Cryo-electron micrograph of the sample analyzed in panel *a* showing SDS-sensitive oligomeric prepores on liposomes (white arrows). Scale bar, 50 nm.

**Figure 5:**
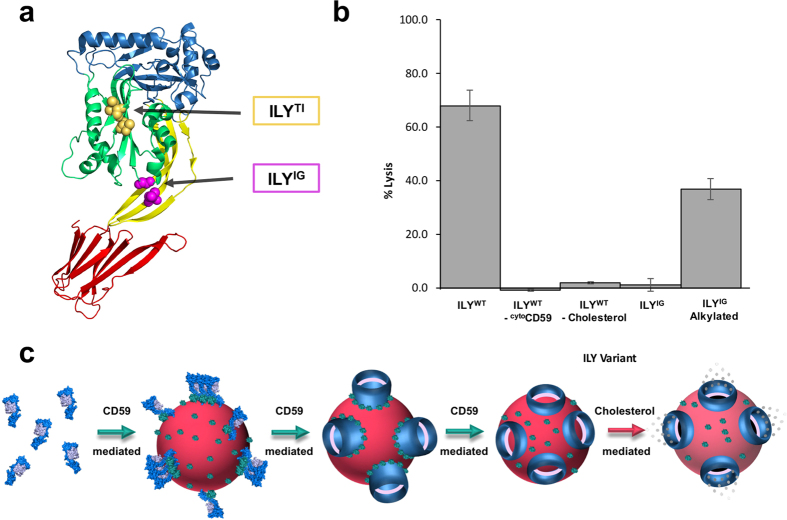
Lytic activity of ILY variants. (**a**) ILY crystal structure (PDB ID: 1S3R)^9^ with domains colored as in Fig. 2. Residues mutated in ILY variants shown as spheres. (**b**) ILY variants were incubated with calcein-containing liposomes decorated with ^cyto^CD59, unless otherwise indicated by *–*^*cyto*^*CD59*. All liposomes contain cholesterol unless otherwise indicated by *–Cholesterol*. Fluorescence measurements, expressed as a percent total lysis, were normalized against background and detergent burst vesicles. Error bars indicate standard deviations across three independent experiments. (**c**) Schematic illustrating the temporal roles of CD59 and cholesterol in ILY pore formation. ILY and CD59 are represented as space-filled models from the crystal structure of the complex (PDB ID: 4BIK). ILY D3 is in lilac; the remainder of the structure is in blue; CD59 is green. Soluble ILY is targeted to cholesterol-containing membranes (red sphere), whereby CD59 sets a specific geometry that defines the diameter of an oligomeric ILY prepore (blue 3D barrel). CD59 triggers the vertical collapse of this prepore, enabling the transmembrane segments of D3 (lilac) to approach the bilayer. CD59 is released from an SDS-resistant late prepore prior to membrane insertion, while cholesterol is required for the final membrane perforation step.

To probe which residues of ILY are essential for CD59 release, a novel prepore state was captured with a disulfide-locked mutant (ILY^IG^) predicted to restrict movement between D2 and D3 (Figs 4 and 5a). Specifically, it locks the base of transmembrane segment 1 (TMS1) of D3 with the adjacent β-strand of D2. Liposome flotation assays of ILY^IG^ confirm the association of ^cyto^CD59-decorated liposomes with an SDS-sensitive prepore (Fig. 4a). Intriguingly, little to no monomeric ILY was observed at the bottom of the gradient. 2D cryo-EM images of ILY^IG^ on ^cyto^CD59–decorated liposomes reveal arc and ring structures on the membrane surface (Fig. 4b). Together these data demonstrate that movement between D3 and D2 is essential for formation of a late prepore intermediate and the release of CD59.

### Probing the trigger for membrane penetration

To gain insight into the molecular mechanism by which the TMSs of ILY insert into the lipid bilayer, we tested the role of cholesterol and of ILY domain mobility using disulfide-locked mutants in a fluorescence-based liposome lysis assay (Fig. 5 and Supplementary Table S2). Fluorescence intensity of calcein released from liposomes upon lysis, relative to the total dye released upon detergent solublization, was used to determine the extent of pore formation. While lysis was observed when wildtype ILY was incubated with cholesterol-containing liposomes decorated with ^cyto^CD59, the absence of either component abolished activity. These data, combined with evidence that CD59 can induce both oligomerization and collapse of a late prepore state, suggest that the role of cholesterol maybe restricted to membrane insertion. Furthermore, lytic activity was abrogated for the novel early prepore state captured by the ILY^IG^ mutant. Alkylation of ILY^IG^ irreversibly reduced its disulfide bond and rescued its activity, confirming that impaired functionality of the mutant is specifically due to the disulfide bond.

## Discussion

CDC pore formation is a complex and co-operative processes involving membrane recognition, oligomerization of monomers, vertical collapse of a prepore, and penetration of the lipid bilayer. For CDCs such as ILY, that require human CD59 in addition to cholesterol to form pores, structural transitions triggered by each component remain less clear. We establish here that CD59 is required for the specific coordination of an ILY oligomer and for the vertical collapse of a late prepore state. We demonstrate that movement of D2 relative to D3 is essential for generating an SDS-resistant late prepore. Furthermore, our results place CD59 release along a timeline of structural transitions for ILY pore formation and reveal that the role of cholesterol may be restricted to the final stages of membrane insertion (Fig. 5c).

Membrane targeting by a receptor is allosterically coupled to oligomerization of CDC monomers. For non-CD59-binding CDCs, the interaction of cholesterol with residues in D4 activates membrane-bound monomers and triggers oligomerization within D1 and D3^23^. Disengagement of the β-5 strand of the central MACPF renders its 4^th^ β -strand accessible to an adjacent monomer^24^. Cholesterol-binding and formation of a stable dimeric intermediate lowers the energy barrier of this normal thermal fluctuation and enables the π-stacking of two key aromatic residues on respective strands to form an SDS-resistant oligomeric prepore^24^. The crystal structure of soluble CD59 in complex with an ILY variant unable to release β-5 from β-4 (ILY^TI^) showed that CD59 coordinates two ILY monomers^19^, reminiscent of the stable dimer formed in the non-CD59-binding cytolysins^25^. In contrast to studies on cells^13^, we observe oligomerization in the absence of CD59 on cholesterol-containing liposomes and lipid monolayers. However, oligomers on monolayers differ from the regular ring and arc-like geometries observed when CD59 is present. Differences observed between model membranes and cells may reflect variations in experimental procedures or the distribution of cholesterol found with these membranes. It is known that that the percentage of cholesterol in the lipid composition and the lipid environment itself, which influences the accessibility of cholesterol’s head group, play key roles in the rate of allosteric activation^26^.

CD59-binding of ILY monomers primes the toxin for integration into an oligomeric prepore. Oligomerization of activated CDC monomers is thought to increase the avidity of the interaction^27^. The lack of unbound monomers observed for the ILY^IG^ prepore-locked mutant further supports this hypothesis. ILY^IG^, free to rotate its β-5 strand, oligomerizes through the edge-on β-sheet extension of the MACPF domain. However this variant, restricted in its mobility of D2 relative to D3, is unable to complete the transition to an SDS-resistant late prepore.

Membrane binding, mediated by cholesterol or CD59, orients CDC monomers nearly perpendicular to the lipid bilayer^7^,^19^,^28^. As a result, the TMSs within D3 are suspended above the target membrane. A vertical collapse of the prepore is necessary to enable membrane penetration of the newly formed hairpins. Here we show that CD59-binding, rather than cholesterol, triggers structural transitions that enable vertical collapse of a prepore.

In summary our findings provide important mechanistic insight into how CD59 and cholesterol coordinate structural transitions of ILY pore formation. Our experiments allow us to understand the temporal relationship of each receptor in forming prepore and pore states. As ILY is an archetypal member of a CDC subset that requires human CD59 for lysis, these data may represent a general molecular mechanism of pore formation for other CD59-binding CDCs.

## Methods

### Expression and purification of CD59 and ILY variants

The extracellular domain of CD59 with an additional C-terminal cysteine residue was expressed in *E.coli* and purified from inclusion bodies as described previously^29^. The cytotopic variant of CD59 used to decorate liposomes, monolayers and supported lipid bilayers was generated using the cytotopic modification reagent bis-myristoyl lysyl SSKKSPSKKDDKKPGD (S-2-thiopyridyl)-cysteine acid (APT3146, Cambridge Research Biochemicals) and purified by hydrophobic interaction chromatography and ammonium sulfate precipitation, as previously described^19^. An *ily* gene lacking cysteine residues (referred to as wildtype ILY) and disulfide-locked *ily* mutants containing the substitutions T346C and I361C (ILY^TI^) and I104C and G244C (ILY^IG^) were expressed in BL21 or Shuffle T7 *E. coli* cells (New England Biolabs) using pTrcHisA vectors. Pairs of residues that could potentially form disulfide bonds were identified using SSBOND^30^. Mutations were introduced using the Quikchange method (Strategene) and confirmed by DNA sequencing. Lack of free sulfhydryl groups from unpaired cysteines was verified using the Thiol and Sulfide Quantitation Kit (Molecular Probes) (Supplementary Fig. S5a). Production of the chromophore p-nitroaniline was measured on a Jenway 7300 spectrophotometer at an absorbance 410 nm. Wildtype ILY and ILY^TI^ were generous gifts from R. Tweten.

Cells were grown to OD_600_ 0.8 at 37 °C and expression was induced with 0.5 mM IPTG overnight at 18 °C. Cultures were pelleted and lysed by sonication in Buffer A (200 mM NaCl, 20 mM Tris-HCl, pH 7.5) containing cOmplete Protease Inhibitors (Roche) and DNAseI (Sigma). His-tagged proteins were bound to cobalt-chelated TALON beads (Clontech), washed with 10 mM imidazole, and eluted in steps of 100 and 500 mM imidazole in Buffer A. Further purification was carried out using size exclusion chromatography in Buffer A on a Superdex 200 10/300 column (GE Healthcare) (Fig. S5b). Purity was assessed by sodium dodecyl sulphide – polyacrylamide gel electrophoresis (SDS-PAGE); proteins detected with Quick Coomassie Stain (Generon).

### Reduction and Alkylation of Disulphide-locked Mutants

ILY^IG^ was irreversibly reduced and alkylated at room temperature using the ProteoPrep^®^ Reduction and Alkylation Kit (Sigma). Briefly, purified protein was exchanged into Buffer A at pH 8.5 and was concentrated to 5 mg/ml. Incubation of the protein with 5 mM tributylphosphine (TBP) for 30 minutes reduced the disulfide bond. Subsequent alkylation was performed by reaction with 15 mM iodoacetamide (IAA) for 1 hour. Excess IAA was quenched with 2.5% (v/v) TBP for 15 minutes. Alkylated protein was spun for 5 minutes at 20,000× g, followed by buffer exchange into Buffer A at pH 7.5.

### Liposome Preparation

1,2-dioleoyl-sn-glycero-3-phospho-L-serine (DOPS) (Avanti Polar Lipids), 1,2-dioleoyl-sn-glycero-3-phosphocholine (DOPC) (Anatrace), 1,2-dioleoyl-sn-glycero-3-phosphoethanolamine (DOPE) (Avanti Polar Lipids) and cholesterol (Sigma) were dissolved in chloroform and mixed (3:2:3:2 w/w ratio). The lipid mix was dried under nitrogen gas and rehydrated in Buffer A. Liposomes were extruded through a 100 nm polycarbonate membrane (Whatman) to create a monodisperse unilamellar liposome population.

### SDS-AGE and Western Blot Analysis

ILY (0.85 μM) was incubated with or without liposomes (0.29 mg/ml) and with or without ^cyto^CD59 (0.85 μM) for 1 hour at 37 °C. Samples were analyzed by SDS-AGE using a 2% (w/v) gel (100 V, 1 hour). The proteins were transferred to a nitrocellulose paper using a TE77 Semi-dry transfer unit (Hoefer) at 70 mA for 2 hours at 4 °C. His-tagged ILY was detected using a Penta-His antibody (Qiagen), followed by a Anti-Mouse IgG -Alkaline Phosphatase (Sigma). Band visualization was carried out using Sigma Fast BCIP/NBT tablets (Sigma).

### Liposome Flotation Assay

Liposomes were tracked by the incorporation of a phosphatidylethanolamine with lissamine rhodamine B-labeled head groups (Avanti Polar Lipids) at a final concentration of 0.1% (w/w) into the lipid mixture. Extruded liposomes at a concentration of 1.5 mg/ml were incubated with ^cyto^CD59 and ILY variants at a molar ratio of 1:1 (4.4 μM) for 1 hour at 37 °C. Samples were mixed with 40% ficoll in Buffer A, resulting in a final ficoll concentration of 20% (w/v). A 10% (w/v) ficoll solution was layered on top, followed by Buffer A alone, creating a step gradient. Gradients were centrifuged using a TLA 120.1 rotor in an OptimaTM Max bench top Ultracentrifuge (Beckman Coulter) at 95, 000 rpm, 4 °C for 1 hour. Gradients were fractionated and analyzed by SDS-AGE and western blotting.

### Fluorescence-Based Liposome Lysis Assay

Lipid films were rehydrated in Buffer A containing 50 mM calcein. Excess calcein was separated from liposomes by gel filtration (Sephadex G-50 beads (Sigma) equilibrated in Buffer A with 500 mM sucrose). Fluorescence intensity was measured using a Varian Cary Eclipse Fluorescence Spectrophotometer (Agilent Technologies) with excitation and emission wavelengths of 490 nm and 520 nm, respectively and a 5 nm slit width. The spectrophotometer was operated in kinetics mode and readings were recorded every 30 seconds. ^cyto^CD59 (0.5 μM) was incubated with 190 μl of fluorescently-labeled liposomes and the average background fluorescence intensity was measured for 10 minutes. Subsequently, ILY variants (0.5 μM) were added, incubated for 1 hour at 37 °C, and fluorescence was measured for an additional hour. Liposomes were burst by the addition of 0.2 M C_12_E_8_ detergent (Sigma) and the maximum fluorescence per reaction was measured for a further 10 minutes. Fluorescence measurements were normalized to the background and detergent readings for each reaction.

### Preparation and Negative Staining of Lipid Monolayers

Monolayers were assembled in a Teflon plate containing wells. ILY and ^cyto^CD59 were incubated at 37 °C in Buffer A and overlaid with 2 μl of chloroform solution containing 1 mg/ml lipid. For the lipid composition DOPC:cholesterol:DOPS (5:4:1 w/w ratio), ILY was mixed with ^cyto^CD59 in an equimolar amount (0.169 μM) for 30 minutes. For samples lacking ^cyto^CD59, ILY (0.127 μΜ) was incubated with lipids for 1 hour. Monolayers lacking cholesterol (DOPC:DOPS, 9:1 w/w ratio), were incubated with equimolar ILY and ^cyto^CD59 (0.169 μM) for 5 minutes. Copper grids with a continuous carbon film (Electron Microscopy Sciences) were applied to the top of wells. The plate was sealed in a petri dish containing tissue soaked in Buffer A. Grids were removed and samples negatively stained with 2% (w/v) uranyl acetate. Monolayers containing cholesterol were imaged on a Tecnai 12 (LaB6 filament) at 120 kV with a magnification of 30, 000× (4.48 Å/pixel) or 52,000× (2.6 Å/pixel). Images were acquired using a 2 k × 2 k CCD camera (F216, TVIPS). Monolayers lacking cholesterol were imaged on a Tecnai G2 Spirit BioTWIN (Tungsten filament) at 120 kV with magnification of 52, 000× (4.35 Å/pixel). Images were acquired using a 2 k × 2 k Eagle CCD camera (FEI).

### Cryo-Electron Microscopy

Equimolar ILY and CD59 (2.2 μM) were incubated with 0.75 mg/ml liposomes for 1 hour at 37 °C. Samples (2.5 μl) were applied to glow discharged Quantifoil R2/2 holey carbon grids and plunge frozen in liquid ethane cooled to liquid nitrogen temperature using a Mark III vitrobot (FEI). Images were acquired on a Philips CM200 (FEG) microscope, 4 k × 4 k TemCam-F415MP CCD camera at 50, 000× magnification (1.76 Å/pixel) under low dose conditions. The defocus range used was 2–4 μm under focus.

### Atomic Force Microscopy

Supported lipid bilayers of DOPC: cholesterol:DOPS (5:4:1 molar ratio) and DOPC:DOPS (9:1 molar ratio) were prepared via the vesicle fusion method^31^. Briefly, a dried lipid film was rehydrated in Buffer A and vortexed to generate a liposome suspension. The resulting suspension was sonicated to form small unilamellar vesicles and incubated upon freshly cleaved mica in the presence of 5 mM calcium chloride for 20 minutes at room temperature. Supported lipid bilayers were rinsed with Buffer A to remove excess vesicles from the supernatant. Equimolar ILY:CD59 (0.17 μM) was added to supported bilayers and incubated at 37 °C. Measurements were recorded at room temperature on a MultiMode IV (Bruker) operated in tapping mode at a resonance frequency of 7–9 kHz, using PNP-TR and BioTool tips (Nanosensors) and with an ‘E’ scanner. Lines were recorded at 1 Hz and image analysis was carried out using Nanoscope Analysis 1.5 (Bruker).

## Additional Information

**How to cite this article**: Boyd, C. M. *et al*. Disentangling the roles of cholesterol and CD59 in intermedilysin pore formation. *Sci. Rep.* **6**, 38446; doi: 10.1038/srep38446 (2016).

**Publisher's note:** Springer Nature remains neutral with regard to jurisdictional claims in published maps and institutional affiliations.

## Supplementary Material

Supplementary Information
