# Supplementary material for: Disentangling the roles of cholesterol and CD59 in intermedilysin pore formation
Source: Sci Rep. 2016 Dec 2;6:38446. doi: 10.1038/srep38446 (PMC5133593; doi:10.1038/srep38446)
Supplement: Supplementary Information [file srep38446-s1.pdf]

Disentangling the roles of cholesterol and CD59

in intermedilysin pore formation

Courtney Boyd [1], Edward S. Parsons [2,3], Richard A. G. Smith [4], John M. Seddon [2], Oscar Ces [2], Doryen Bubeck [1]*

1 Department of Life Sciences, Sir Ernst Chain Building, Imperial College London, London SW7 2AZ, UK

[2] Department of Chemistry and Institute of Chemical Biology, Imperial College London, London SW7 2AZ, UK

[3] London Centre for Nanotechnology, University College London, London WC1H 0AH, UK

[4] MRC Centre for Transplantation, King's College London, 5th Floor Tower Wing, Guys' Hospital, London SE1 9RT, UK

*To whom correspondence should be addressed

^{cyto}CD59	+	+	-
**Cholesterol**	+	-	+
**Mean Diameter (Å)**	385.7	382.2	402
**Standard Deviation**	22.7	18.4	34.9

Supplementary Table S1 Outer diameter dimensions of circular ILY oligomers formed on negatively-stained lipid monolayers. The + or − denoted at the top of the table indicates whether the toxin was incubated with or without cholesterol-containing liposomes or ^{cyto}CD59. A total of 50 oligomers were measured for each condition using ImageJ[1].

	**3:2:3:2 DOPS:DOPC:DOPE:Cholesterol**		**% Lytic Activity**	**Standard Deviation**
**ILY Variant**	**CD59**	**Cholesterol**		
ILY^{WT}	+	+	67.9	5.7
ILY^{WT}	-	+	0.0	0.3
ILY^{WT}	+	-	1.7	0.4
ILY^{WT}	-	-	1.1	0.6
ILY^{IG}	+	+	0.9	2.4
ILY^{IG} Alkylated	+	+	36.9	4.0
**5:4:1 DOPC:Cholesterol:DOPS**				
ILY^{WT}	+	+	69.1	0.7

Supplementary Table S2 ILY variants (0.5 µM) were incubated with calcein-containing liposomes under various conditions; in the presence or absence of ^{cyto}CD59 and/or cholesterol as detailed. Lysis was observed only in the presence of both ^{cyto}CD59 and cholesterol, indicating the absolute requirement for both to be present for ILY lytic activity. Equivalent activity was observed over lipid compositions used for both

liposome (3:2:3:2 wt/wt ;DOPS:DOPC:DOPE:Cholesterol) and planar monolayer/bilayer (5:4:1 wt/wt; DOPC:Cholesterol:DOPS) experiments. Lytic activity of ILY^{IG} mutant was successfully reconstituted on irreversible reduction and alkylation of its disulfide bond. Values indicate the mean % lysis and standard deviation across three independent experiments.

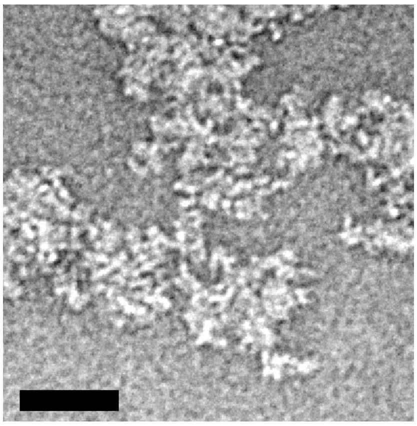

Supplementary Figure S1 The disulfide-locked mutant ILY^{TI} (0.127 µM) forms large, non-specific aggregates on negatively-stained lipid monolayers containing equimolar ^{cyto}CD59, akin to those observed by AFM on supported lipid bilayers. Scale bar, 50nm.

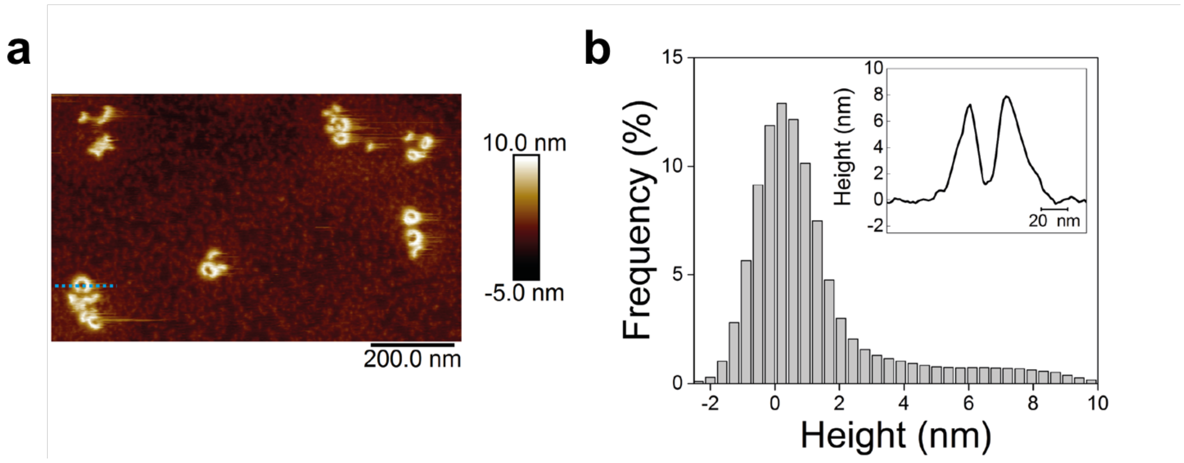

Supplementary Figure S2 Low surface coverage of ILY pores was obtained by incubating equimolar ILY and ^{cyto}CD59 (0.127 μM) with cholesterol-containing supported lipid bilayers. AFM image **(a)** shows the exposed bare membrane (darkest patches), molecules of ^{cyto}CD59 (lighter punctate spots), and ILY pores (brightest intensity arcs and rings). Color scale, 15 nm. **(b)** Height data, plotted as a histogram of the z measurements for each pixel in the image; inset shows a cross-section of the area highlighted by the blue dotted line in *a*.

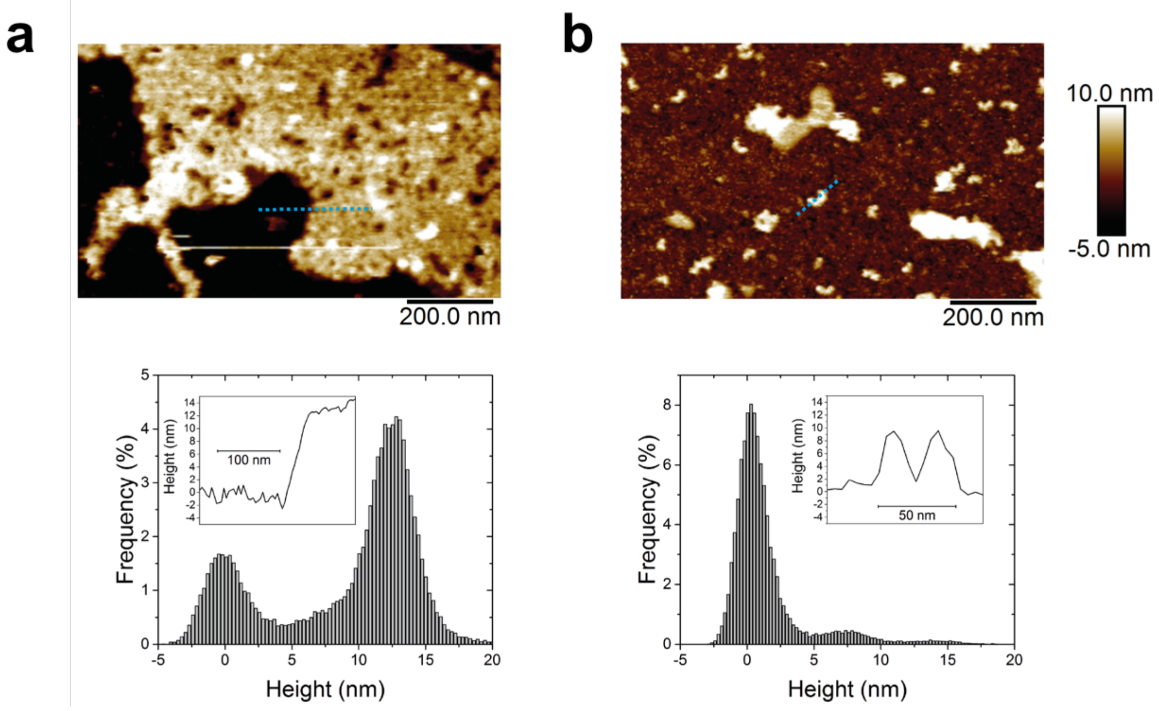

Supplementary Figure S3 Cholesterol alone is insufficient to trigger collapse of the ILY prepore. **(a-b)** AFM images (top panel) and corresponding height data (bottom panel) for ILY prepores formed on cholesterol-containing supported lipid bilayers in the absence of ^{cyto}CD59. Similar to negative stain images of monolayers as seen in *Figure 1d* **(a)** shows large aggregated patches of prepore oligomers while **(b)** shows the more common smaller aggregates. Height data is plotted as a histogram of the z measurements for each pixel in the image; inset shows a cross-section of the area highlighted by the blue dotted line. Color scale, 15 nm.

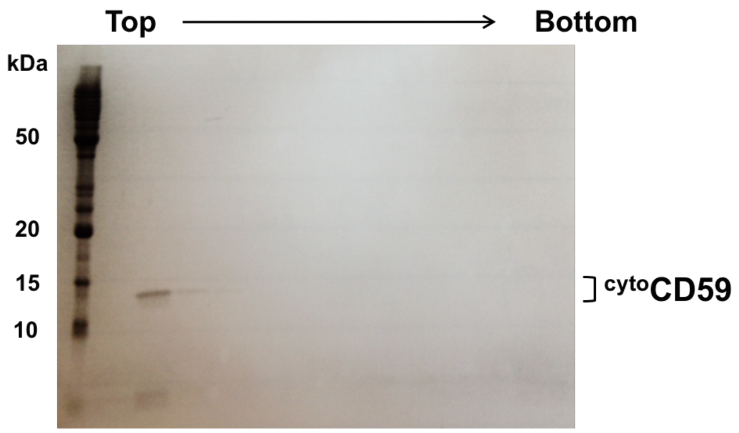

Supplementary Figure S4 ^{cyto}CD59 was incubated with rhodamine-labeled liposomes and subjected to density centrifugation through ficoll. Gradient fractions that contained protein were identified using SDS-PAGE. ^{cyto}CD59 was only found to be present in lipid-containing fractions at the *Top* of the gradient. No protein was detected in the *Bottom* fractions by Coomasie staining. Molecular weight markers are in the far left lane.

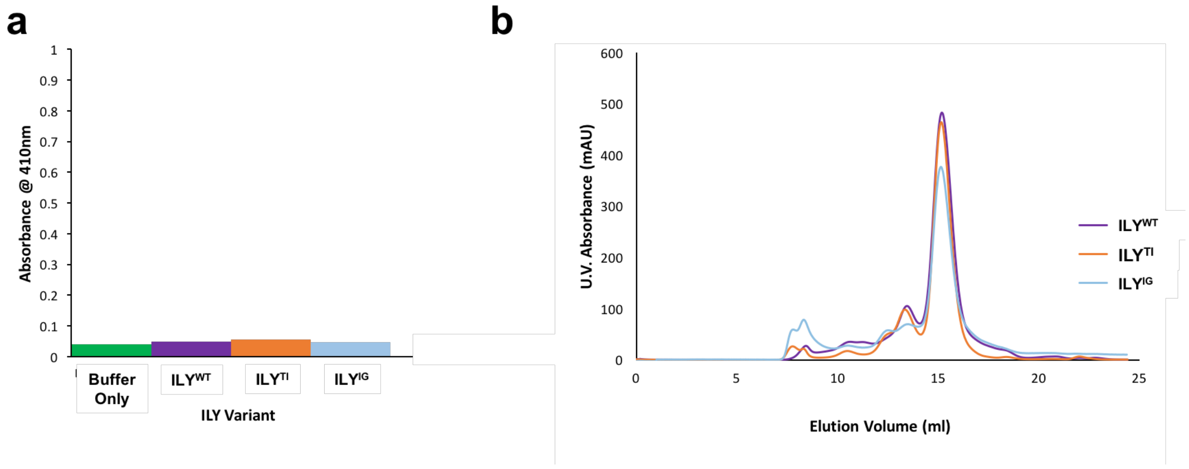

Supplementary Figure S5 ILY variants form intramolecular disulfide bonds. **(a)** The presence of free-thiols, indicating un-paired cysteine residues, was assessed using a chromogenic assay. Disulfide-locked mutants ILY^{TI} and ILY^{IG} displayed no increase in free-thiol content compared to wildtype toxin lacking cysteines. **(b)** Size-exclusion chromatography elution profiles for ILY variants show monodisperse peaks at the same position as wildtype, consistent with correctly paired disulfide-bonds.

Supplementary References

1 Schneider, C. A., Rasband, W. S., Eliceiri, K. W. NIH Image to ImageJ: 25 years of image analysis. *Nature Methods, **9***, 671-675 (2012).
